# Forecasting infectious disease emergence subject to seasonal forcing

**DOI:** 10.1186/s12976-017-0063-8

**Published:** 2017-09-06

**Authors:** Paige B. Miller, Eamon B. O’Dea, Pejman Rohani, John M. Drake

**Affiliations:** 1 University of Georgia, Odum School of Ecology, 140 E. Green Street, Athens, USA; 2 Center for the Ecology of Infectious Diseases, University of Georgia, Athens, USA; 3 Department of Infectious Diseases, University of Georgia, Athens, USA

**Keywords:** Disease forecasting, Seasonality, Critical transition

## Abstract

**Background:**

Despite high vaccination coverage, many childhood infections pose a growing threat to human populations. Accurate disease forecasting would be of tremendous value to public health. Forecasting disease emergence using early warning signals (EWS) is possible in non-seasonal models of infectious diseases. Here, we assessed whether EWS also anticipate disease emergence in seasonal models.

**Methods:**

We simulated the dynamics of an immunizing infectious pathogen approaching the tipping point to disease endemicity. To explore the effect of seasonality on the reliability of early warning statistics, we varied the amplitude of fluctuations around the average transmission. We proposed and analyzed two new early warning signals based on the wavelet spectrum. We measured the reliability of the early warning signals depending on the strength of their trend preceding the tipping point and then calculated the Area Under the Curve (AUC) statistic.

**Results:**

Early warning signals were reliable when disease transmission was subject to seasonal forcing. Wavelet-based early warning signals were as reliable as other conventional early warning signals. We found that removing seasonal trends, prior to analysis, did not improve early warning statistics uniformly.

**Conclusions:**

Early warning signals anticipate the onset of critical transitions for infectious diseases which are subject to seasonal forcing. Wavelet-based early warning statistics can also be used to forecast infectious disease.

**Electronic supplementary material:**

The online version of this article (doi:10.1186/s12976-017-0063-8) contains supplementary material, which is available to authorized users.

## Background

Improvement of methods for forecasting infectious disease dynamics is of tremendous value to public health and the global economy [1]. Disease forecasting is challenging because transmission is subject to complex interactions among a variety of independent actors, non-linearity, noise, and seasonality driven by age [2] and spatial structure [3], susceptible depletion [4, 5], environmental variability [6], and behavior [5, 7]. Despite these challenges, a number of approaches have been developed along distinctly different lines: (i) sequential Monte-Carlo methods (e.g., [8]), (ii) data assimilation techniques inspired by numerical weather prediction [9, 10], and (iii) “Wisdom of the Crowd” approaches [11] among others. Monte-Carlo methods aim to estimate unobserved states in Markov chains (such as the size of the infectious population) while simultaneously estimating unknown parameters [8]. In contrast, ensemble Kalman adjusted filters are commonly used in data assimilation and take advantage of incoming information by iteratively updating an ensemble of models to contract variance [9, 10]. Lastly, “Wisdom of the Crowd” approaches incorporate experts’ experience and knowledge [11]. These studies demonstrate that accurately forecasting the trajectory of infectious disease incidence is possible. In contrast, we are in the earlier stages of theory and method development for anticipating the emergence of infectious disease [12].

Emerging infectious diseases of the past century include three major influenza pandemics, Ebola, HIV, and Zika. The increasing frequency of emergence by novel pathogens is usually attributed to changes in socio-economic, environmental and ecological factors [13]. All of these are now combated with highly developed diagnostics, therapeutics, and behavioral interventions. If they could have been anticipated, behavioral interventions could have been deployed earlier, preventing loss of life and reducing spread even while medical countermeasures were under development. Additionally, some countries have reported resurgence of vaccine preventable diseases (e.g., pertussis, measles) despite high rates of vaccination [14]. Many candidate hypotheses have been advanced to explain the resurgence of vaccine preventable diseases including changing vaccination schedule and composition, changing immune acquisition, and evolution of the pathogen (reviewed in [14]). The economic and health benefits of the increased preparedness that could be achieved by more advanced warning of disease emergence and re-emergence would be substantial.

In the past decade, researchers have studied an alternative, model-independent forecasting approach which summarizes the patterns of fluctuations in a system to infer whether it is close to a critical transition [15–18]. A critical transition is a sudden and large change in the state of a dynamical system [19]. Statistical signals primarily result from a phenomenon known as *critical slowing down* (CSD), which is a signature of a second-order phase transition and refers to the increased relaxation time for a near-critical system to reach equilibrium following a perturbation compared with a system that is far from critical [20]. Critical slowing down occurs in a wide range of natural tipping points, including large-scale climate shifts [15], population collapse [17], and lake eutrophication [21]. O’Regan and Drake [22] proposed that statistical early warning signals (EWS) may also precede the onset of sustained transmission in a contagion process where the approach to disease emergence is marked by a slow drift to criticality. Because of the key assumption of slow drift, critical slowing down has primarily been considered in non-periodic systems [23]. But, many diseases exhibit periodic forcing due to seasonal variations in climate [24, 25], human behavior [26], or immune function [27]. Because seasonal forcing results in periodic sojourns near criticality, we wondered if standard approaches to detecting critical slowing down might therefore fail in systems such as resurgent childhood infections forced by the annual calendar of school terms [5, 28].

We performed a simulation study to investigate how periodic forcing might interfere with the detection of critical slowing down in directly-transmitted disease systems. A recent review of resilience in ecological systems noted that early warning signals are difficult to detect in systems with intrinsic cycles [23]. In this case, [23] recommend first removing periodic trends in the time series through seasonal detrending and then performing analysis on the residuals. However, prior to reaching criticality, transmission systems may not exhibit a reliably periodic pattern. Thus, it’s possible that periodic detrending or differencing may corrupt the signal of CSD and unintentionally reduce the reliability of the early warning signals. Nonetheless, the robustness of differencing and detrending have never been validated in the context of early warning signals of epidemic transitions. To fill this gap, we studied the detectability of CSD prior to disease emergence in simulations. To address the issue of periodic forcing, we analyzed our data both with and without first removing seasonal trends via seasonal detrending and differencing.

Additionally, we propose and examine two new statistics based on components of the frequency domain as alternatives to seasonal detrending and differencing. In the frequency domain of time series without periodic cycles, CSD manifests as spectral reddening, or the dominance of low frequencies prior to a transition [29]. Spectral reddening of the Fourier spectra was shown to be a reliable indicator of climate tipping points [29] and housing market changes [30, 31]. Our concern is that for a periodic time series, the periodic signal will overwhelm that of spectral reddening such that CSD is difficult to detect. Wavelet analysis, a non-parametric approach to identifying periodic components of the system over time, might allow us to identify the frequencies that are most sensitive to CSD. In this paper, we show that signals of CSD can still be found in systems with periodic behavior and present methods for forecasting emergence of seasonally forced infectious diseases based on spectral reddening of the wavelet spectrum.

## Methods

### SIR model

To simulate the dynamics of an immunizing pathogen with seasonal transmission (e.g., pertussis, measles) [7], we considered a seasonally-forced stochastic Susceptible-Infected-Recovered (SIR) model. Transitions between states occur with rates shown in Table 1 where *N*=*S*+*I*+*R* and the mean field equations are

1 $$\begin{array}{@{}rcl@{}} \frac{d(S/N)}{dt} &=& -\beta(t) (S/N)(I/N)-\xi(S/N)+\mu(1-S/N)  \\ \frac{d(I/N)}{dt} &=& \beta(t) (S/N)(I/N)+\xi (S/N)-\gamma (I/N)-\mu (I/N) \\ \frac{d(R/N)}{dt} &=& \gamma (I/N)-\mu (R/N) . \end{array} $$

**Table 1:** Transition rules for stochastic SIR model

Event	(*Δ* *S*,*Δ* *I*,*Δ* *R*)	Rate
Birth of a susceptible	(1,0,0)	*μ* *N*_0_
Death of a susceptible	(−1,0,0)	*μ* *S*
Infection	(−1,1,0)	$\frac {\beta S I}{N} + \xi S$
Death of an infective	(0,−1,0)	*μ* *I*
Recovery of an infective	(0,−1,1)	*γ* *I*
Death of a removed	(0,0,−1)	*μ* *R*

Parameter definitions and values are given in Table 2. We modeled the contagion process as a Markov chain using Gillespie’s direct method [32]. A Markov chain is a stochastic process with the property that the future state of the system is dependent only on the present state of the system and conditionally independent of all past states (i.e., the system has a memoryless property).

**Table 2:** SIR model parameters

Symbol	Definition	Value
*β*	Transmission rate	varied
*ξ*	Importation rate	6·10^−5^ per year
1/*γ*	Infectious period	14 days
1/*μ*	Lifespan	70 years
*N*_0_	Initial population size	2·10^5^ individuals

Our model is slowly forced through a transition from subcritical (average *R*_0_ during a year less than 1) to supercritical (average *R*_0_ during a year greater than 1) by linearly increasing the average transmission rate, *β*_0_, over time. Increasing transmission, as formulated in this model, can be envisioned as changes in behavior between individuals in a population leading to higher contact rates over time or pathogen adaptation. We assumed frequency-dependent transmission but note that in this case, the difference between density-dependent and frequency-dependent transmission is negligible because the population size fluctuates minimally (due to demographic stochasticity).

**Fig. 1:**
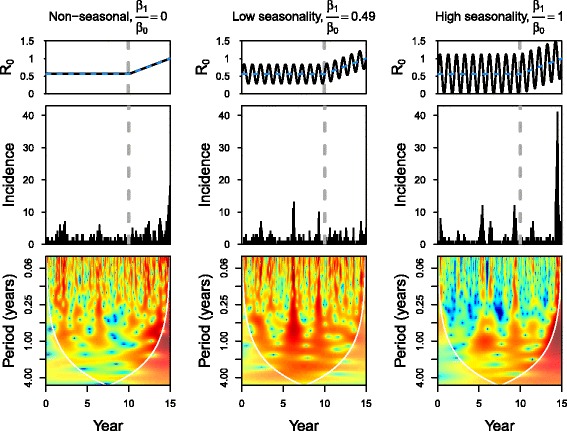
We simulated a directly transmitted immunizing pathogen with 50 varying intensities of seasonal transmission and then determined if signatures of critical slowing down were detectable. In all simulations, average *R*_0_ (i.e., the number of secondary infected cases arising from a single infected case in an entirely susceptible population) increases linearly after year 10 (*t*_10_) and fluctuates seasonally according to the amplitude of seasonality, *β*_1_. From left to right, *β*_1_ is equal to 0, 0.019, 0.04 and *β*_0_=0.04/ day. The amplitude relative to baseline transmission (referred to here as *relative fluctuation*) are equal to 0, 0.49, and 1. Values of other parameters are given in Table 2. In the top two rows, a grey dashed line divides the time series into its null (left of the grey line) and test (right of the grey line) intervals used for calculating reliability of each early warning statistic. The bottom row shows the wavelet power spectrum of the simulated data (middle row) with time along the x-axis and frequency along the y-axis. Lower frequencies correspond to longer periods (i.e., the bottom of the spectrum) and higher frequencies correspond to lower periods (i.e., the top of the spectrum). The colors code for power coefficients from dark blue, low values (0), to dark red, high values (1·10^5^). High wavelet coefficients indicate which frequencies are more powerful at that point in time

In our model, susceptible individuals are infected through contact with infected individuals within the population at a time-varying rate *β*(*t*). To represent a realistic scenario such that low levels of contact with outside populations also occur, we assumed that susceptible individuals can also became infected at a constant rate *ξ* [22]. This assumption signifies that the equilibrium size of the infectious population is non-zero, allowing for stuttered chains of transmission even when the system is sub-critical (i.e., when *R*_0_<1). Sinusoidal transmission follows the function of time *β*(*t*)=*β*_0_(*t*)+*β*_1_ sin(2*π* *t*/365) where *t* is in units of days. We assume the time-localized average rate of transmission (*β*_0_) begins to increase linearly ten years later (i.e., *t*_start_=10·365 days), so that

2 $$\begin{array}{@{}rcl@{}} \beta_{0}(t)= \left\{ \begin{array}{ll} \beta_{k}, & t < t_{\text{start}} \\ \beta_{k} + b\left(t-t_{\text{start}}\right), & t \geq t_{\text{start}} \end{array}\right. \end{array} $$

where *β*_*k*_=0.04 is the initial transmissibility and *b*=1.7242×10^−5^/day is the daily rate at which transmission increases. These parameters cause the critical value of *R*_0_ to be reached at year fifteen (five years after *t*_start_). Before *t*_start_, the average *R*_0_, calculated as *β*/(*γ*+*μ*), is approximately 0.56 (*β*_0_=0.04). To explore the effect of seasonality, we varied *β*_1_ linearly from 0 to 0.04 with 50 levels and simulated each level 250 times. For sinusoidal seasonal forcing, we present and discuss intensity of *relative fluctuations*, i.e. *β*_1_/*β*_0_, for ease of interpretation. For example, if *β*_1_/*β*_0_=1 then seasonality would cause *R*_0_ to fluctuate between 0 and twice that of baseline transmission (Fig. 1).

We note that seasonality in transmission is often modeled as *β*=*β*_0_(1+*β*_1_ cos(2*π* *t*/*p*)) where *β*_1_ can be interpreted as the amplitude of fluctuations relative to the average rate of transmission (see [33]). In this formulation, though, if *β*_0_ were to increase (i.e., as formulated in our model) then the size of oscillations around the increasing average transmission, *β*_0_, would increase as well. By contrast, our parameterization induces a constant amplitude for seasonality. The relative amplitude of fluctuation in our parameterization is simply given by *β*_1_/*β*_0_.

For validation of our results with sinusoidal forcing, we also analyzed the impact of seasonal transmission on EWS using square wave forcing, term-time forcing [28], and monthly averaged rates [5]. These other seasonality functions were defined by a set of 52 repeating deviations from the mean. The magnitude of seasonality for these models was controlled by scaling the deviations such that the deviation below the mean was at most equal to a given fraction of the mean.

We allowed a burn-in time of ten years before analyzing simulated incidence to remove the effects of transient behavior. For comparison with typical incidence data, cases were aggregated as number of individuals entering the removed class each week denoted by *X*_t_. We assumed no under-reporting. Because we assumed individuals within the population could become infected from those outside the population, sparking transmission with initially infected individuals was not necessary. Thus, our initial conditions for state variables were *S*(0)=*N*(0), *I*(0)=0, *R*(0)=0. All simulations were performed in R using packages spaero [34] and pomp [35]. Code to reproduce the simulations is available online at https://github.com/project-aero.

### Calculation of early warning signals

We defined the null and test intervals to compare trends in EWS when the system is and is not approaching criticality. The null interval is defined when *β*_0_ remains constant as compared with the test interval when *β*_0_ is increasing and therefore approaching criticality (Fig. 1). Following separation into the null and test intervals, we calculated early warning statistics both with and without first removing seasonal trends via (1) seasonal decomposition by Lowess [36] and (2) seasonal differencing where the time series at *X*_*t*_ is subtracted from *X*_*t*−52_ when observations are measured weekly.

We analyzed two statistics for anticipating disease emergence based on the wavelet spectrum: (1) *wavelet filtered reddening* and (2) *wavelet spectral reddening*. We defined wavelet filtered reddening, $\bar {W}_{t,j_{1} j_{2}}^{2}$, as any quantity proportional to the variance of the time series after it is filtered to include only its components in a wavelet transform’s frequency domain with scales ranging from $s_{j_{1}}$ to $s_{j_{2}}$. We calculated it according to

3 $$ \bar{W}_{t,j_{1} j_{2}}^{2} = \frac{0.34 T \delta_{t} }{\lambda} \sum\limits_{j = j_{1}}^{j_{2}} \frac{|W_{t}(s_{j})|^ 2}{s_{j}},  $$

where *T* is the total number of observations in the times series, *δ*_*t*_ is the difference in time between observations, *s*_*j*_ is a wavelet scale, *W*_*t*_(*s*_*j*_) is a wavelet transform of the observation time series *X* with localized time index *t* and scale *s*_*j*_, and *λ* is the ratio of the Fourier period of the wavelet function to its scale. The Fourier period of the wavelet function is the period at which the Fourier transform of the wavelet function peaks.

To see that (3) is proportional to the average variance of the time series within a frequency band, note that the right hand side of (3) differs from that of Eq. 24 of Ref. [37] by a constant factor only. Equation (3) may also be viewed as a partial summation over elements of the bias-corrected power spectrum of Ref. [38] multiplied by a constant factor. Thus in our plot of the wavelet power spectrum, we plot the values of [0.34*T* *δ*_*t*_|*W*_*t*_(*s*_*j*_)|^2^]/[*s*_*j*_ *λ* *σ* ^2^] for all scales *s*_*j*_ and times *t*, where *σ* ^2^ is the estimated variance of the time series *X*. Because we look at trends in the wavelet power spectrum, the constant factors in our equations have no effect on our results and are included simply because they are included in the calculations of the software package we used.

Next we explain exactly how we calculated the wavelet transform. The wavelet transform was calculated to satisfy

4 $$ W_{t} (s)=\sum\limits_{t'=1}^{T} \left(X_{t'}-\bar{X}\right) \left(\frac{\delta_{t}}{s} \right)^{0.5} \psi_{0}^{*}\left[ \frac{(t' - t) \delta_{t}}{s} \right],  $$

$\bar {X}$ is the mean of the time series, and $\psi _{0}^{*}$ is the complex conjugate of the Morlet wavelet function. The Morlet wavelet function satisfies

5 $$ \psi_{0} (\eta) = \pi^{-1/4} \mathrm{e}^{i \omega_{0} \eta} \mathrm{e}^{-\eta^{2} / 2}  $$

where *ω*_0_ is the nondimensional frequency. We used typical parameters to calculate the wavelet transform. We set *ω*_0_ to 6, and the sequence of scales used started at 2*δ*_*t*_ and increased geometrically with 12 equal steps per octave until it reached about one third the duration of the time series. These parameters correspond to the defaults in the R package biwavelet [39], which we used for all wavelet calculations. To choose the parameters *j*_1_ and *j*_2_ in Eq. (3), we conducted a grid search and used AUC to determine which *j*_1_ and *j*_2_ resulted in good separation of the distributions of $\bar {W}_{t,j_{1} j_{2}}^{2}$ calculated from emergence and non-emergence simulations.

Our second wavelet statistic, wavelet spectral reddening, was defined as the median scale of the wavelet spectral density at a given time index *t*. We denote this statistic as *s*_median_(*t*), and we calculate it according to

6 $$ s_{\text{median}}(t)=\text{min} \left\{s_{j}\,\text{such that}\, \bar{W}_{t,1 j}^{2} / \bar{W}_{t,1 j_{\text{max}}}^{2} > 0.5 \right\},  $$

where the indices of the increasing wavelet scales *s*_*j*_ run from 1 to *j*_max_. A spectral reddening statistic of the Fourier power spectrum was calculated in an analogous manner in Ref. [31]. This statistic is intended to quantify any increasing dominance of the low frequency components of the power spectrum associated with the decreasing stability of an equilibrium.

The other early warning statistics were computed according to the formulas in Table 3, in which the weights $\phantom {\dot {i}\!}w_{t,t'}$ depend on the smoothing kernel and bandwidth. We used both a uniform kernel and a Gaussian kernel. The equation for the weights using the uniform kernel is

7 $$ w_{t,t'} = \left\{ \begin{array}{ll} 1 / N_{t}, &|t - t'| < b, \\ 0, &\text{otherwise}, \end{array}\right.  $$

**Table 3:** Formulas for moving window statistics

Statistic	Formula
Mean_*t*_	$\sum _{t'=1}^{T} w_{t,t'} X_{t'}$
Variance_*t*_	$\sum _{t'=1}^{T} w_{t,t'} (X_{t'}- \text {mean}_{t'})^{2}$
(Variance convexity)_*t*_	variance_*t*_−variance_*t*−1_
Autocovariance_*t*_	$\sum _{t'=(\text {lag} + 1)}^{T} w_{t,t'} (X_{t'} - \text {mean}_{t'}) (X_{t' - \text {lag}} - \text {mean}_{t' -\text {lag}})$
Autocorrelation_*t*_	autocovariance_*t*_/(variance_*t*_×variance_*t*−lag_)^0.5^
(Decay time)_*t*_	−lag/(log min(max(autocorrelation_*t*_,0),1))
(Index of dispersion)_*t*_	variance_*t*_/mean_*t*_
(Coefficient of variation)_*t*_	(variance_*t*_)^0.5^/mean_*t*_
Skewness_*t*_	$\sum _{t'=1}^{T} w_{t,t'} (X_{t'} - \text {mean}_{t'})^{3} / (\text {variance}_{t})^{1.5}$
Kurtosis_*t*_	$\sum _{t'=1}^{T} w_{t,t'} (X_{t'} - \text {mean}_{t'})^{4} / (\text {variance}_{t})^{2}$

where the normalization constant $N_{t}=\sum ^{\min (T, t + b-1)}_{j=\max (1, t-b + 1)} 1$. The equation for the weights using the Gaussian kernel is

8 $$ w_{t,t'} = f(t - t', b) / N_{t}  $$

where *f* satisfies $f(x, b)=\exp \left (-x^{2}/(2 b^{2})\right)/\left [b \sqrt {2 \pi }\right ]$ and $N_{t}=\sum _{t'=1}^{T} f(t-t', b)$.

Following [17, 22, 40, 41] the other early warning statistics were computed according to the formulas in Table 3. Moving window statistics of time series *X* (centered on index *t*) were calculated with a uniform kernel and the bandwidth (parameter b in Eq. 7) was 100. If data were detrended using STL [36], the equations for the moving window statistics were slightly modified. The STL estimate of the trend was used as mean_*t*_ in Table 3. Then, the STL estimate of the irregular component of the time series was substituted in for the residuals (*X*_t_−mean_*t*_) in Table 3.

We determined the performance of each EWS using the AUC statistic as follows. We calculated the association between each indicator time series and time using Kendall’s rank correlation coefficients. Since we had 250 simulations, we generated a distribution of correlation coefficients for each indicator when the system was and was not approaching disease emergence (i.e., from the test or null interval). The amount of overlap between the distributions was calculated with the AUC statistic: AUC=[*r*_test_−(*n*_test_+1)/2]/(*n*_test_ *n*_null_) where *r*_test_ is the sum of the ranks of test coefficients in a combined set of test and null coefficients (where the lower numbers have lower ranks), *n*_test_ is the number of test coefficients, and *n*_null_ is the number of null coefficients. The AUC of an EWS is the probability that a randomly chosen test coefficient is higher than a randomly chosen null coefficient [42].

## Results

**Fig. 2:**
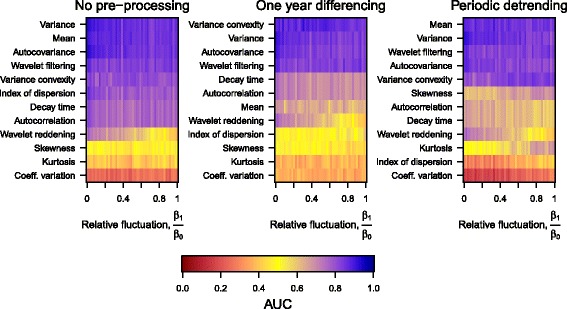
Performance of EWS over a moving window as measured by AUC for SIR systems approaching emergence in the presence of seasonal transmission. The heat plots show the relationship between AUC and seasonality without first removing seasonal trends (left), with one year differenced data (middle), and with seasonal decomposition (right). Data were not pre-processed prior to wavelet-based EWS. In each heat plot, seasonal intensity increases from left to right. The statistics are plotted with highest mean AUC to lowest from top to bottom. The bandwidth for all EWS is equal to 100 weeks (but the bandwidth is not pertinent for wavelet EWS). The AUC value indicates the area under the corresponding ROC curve. An AUC value of 1 indicates perfect detection ability while a value around 0.5 signifies that the statistic is no better than chance at distinguishing between data approaching emergence and data not approaching emergence

**Fig. 3:**
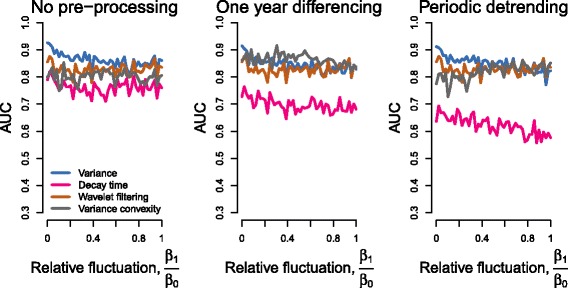
Relationship between increasing seasonal amplitude and reliability of top-performing EWS. Early warning signals remain reliable at the highest level of relative fluctuations (*β*_1_/*β*_0_). Each plot shows the top performing EWS when analysis is performed on raw data (left), yearly differenced data (middle), and residuals following seasonal detrending (right)

We documented signals of critical slowing down when transmission was subject to periodic variation, addressing a current gap in studies of early warning signals for critical transitions (Fig. 2). The AUCs of most early warning statistics were negatively associated with increasing seasonal amplitude (Figs. 2 and 3). When transmission was not subject to seasonal forcing, the most reliable statistics (mean, variance-based, and wavelet filtering), achieved AUCs above 0.85 regardless of how data were pre-processed. For simulations with the highest amplitude of seasonal transmission, AUCs for the mean, variance, autocovariance, and wavelet filtering decreased by 0.05, 0.07, 0.06, and 0.02 compared to the non-seasonal simulations, respectively.

Skewness and kurtosis were poor indicators of disease emergence compared with variance-based statistics but detrending the time series prior to analysis improved the AUCs slightly when transmission was subject to moderate or high levels of seasonal forcing (Right panel, Fig. 2). For other EWS (i.e., decay time, autocorrelation, and index of dispersion), pre-processing the time series prior to analysis worsened or had little impact on the AUCs (Middle and left panels, Fig. 2).

**Fig. 4:**
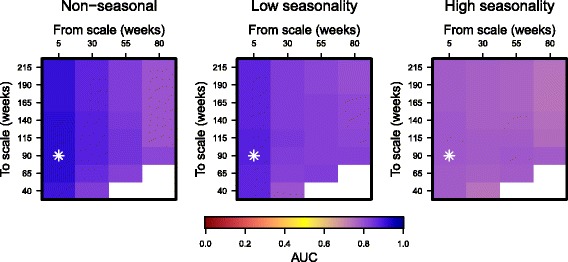
AUC values for filtered wavelet reddening. We calculated filtered reddening by summing Morlet wavelet coefficients within an interval of frequencies over time of the wavelet spectra. The lower and upper bounds for the interval are shown on the top and left sides of each heat plot, respectively. Heat plots from left to right show AUC values for spectral reddening in non-seasonal, moderately seasonal, and highly seasonal (relative fluctuations = 0, 0.5, and 1) transmission. The star represents the filtered reddening band that achieved the highest AUC statistic for forecasting disease emergence for each level of seasonality. We used the band from 30 to 115 weeks in EWS calculations shown in Figs. (2 and 3)

In simulations with non-seasonal transmission, wavelet reddening and filtering performed similarly to variance-based early warning statistics (Figs. 2 and 3). However, as the amplitude of seasonality increased, the AUC of wavelet reddening decreased drastically (from 0.72 to 0.35) with increasing seasonal amplitude whereas the AUCs of wavelet filtering remained relatively constant as seasonal amplitude increased. For all levels of seasonality, the AUCs of wavelet filtering were highest when the frequency band ranged from 5 to 90 weeks (Fig. 4).

Early warning signals performed similarly regardless of the function of seasonal forcing (sinusoidal, term-time [28], monthly averaged [5], and square-wave seasonal forcing) (Additional file 1). For each type of seasonal forcing, the most reliable early warning statistics were variance, variance convexity, autocovariance, and wavelet filtering (Additional file 1). Conventional early warning signals were sensitive to the choice of bandwidth. When the bandwidth was 150 weeks long, rather than 100, early warning signal reliability decreased slightly (Additional file 1). The choice of window shape (i.e., uniform or Gaussian) was less important and resulted in similar AUC values for moving window statistics.

## Discussion

A principal challenge in infectious disease epidemiology is predicting epidemics. Prediction of epidemics is made difficult by a combination of non-linearities driving the system. Early warning signals for disease emergence are potentially useful for predicting emerging infectious diseases because they do not require a detailed understanding of the system’s drivers. For infectious diseases without a seasonal trend (e.g., STDs), generic statistical signals are present before critical transitions [22]. However, seasonal variation alters the spread and persistence of many diseases [33] and has been noted as a limitation to using early warning signals [23]. Understanding how seasonal forcing, a common feature of disease dynamics, affects the trends in generic leading indicators of stability is therefore important for accurately inferring whether a disease is at risk of emergence.

We conducted a simulation study of early warning signals for emergence of infectious diseases. To understand the relationship between seasonality and the predictability of disease emergence, we varied the amplitude of seasonal transmission. We showed numerically that critical slowing down anticipates transitions in periodic systems. Additionally, we proposed and measured the performance of two new leading indicators, wavelet spectral reddening and filtering, compared to conventional early warning signals. Wavelet filtering, calculated by summing specific coefficients of wavelets across the time series, was among the top performing EWS. Our second statistic, wavelet reddening, was less reliable than wavelet filtering as the level of seasonal transmission increased. The advantage to using wavelet-based statistics is that they do not require a choice of bandwidth. Although wavelet filtering requires a choice of frequency band, we showed that the optimal choice of the frequency band was invariant to three levels of seasonal forcing. Furthermore, the AUCs for wavelet filtering were generally highest with the largest frequency bands. This suggests that all periods might be used and therefore this statistic would be parameter-free.

Our study’s purpose was to characterize the impact of seasonality on early warning signals for disease emergence of an immunizing pathogen such as childhood infections. To do this, we varied only the amplitude and held constant the frequency of the periodic component of transmission. We showed numerically that early warning signals can be reliable even in systems with highly seasonal transmission. A recent study [43] proposed special methods for calculating early warning signals when the time scale of the system is similar to that of the forcing. In our model, the time scale of the system could be characterized by the decay time of the number of cases per week and the time scale of the forcing is the period of the seasonal cycle in *R*_0_. To characterize the timescale of the model as a function of *R*_0_, we calculated the ensemble decay time based on the ensemble of *X*_*t*_ comprising the simulation replicates of our model. We used simulations with a relative fluctuations of zero and an initial *β* of 0.04. We found that the estimated decay time averaged 0.017 years in the null interval and rose to 0.14 years over the course of the test interval. Therefore, within the test interval the timescale of the system was similar to that of the annual forcing. There are many differences between our model and the model analyzed in reference [43]. For example, stochastic effects (e.g., imported cases) are more important in our model and the periodic forcing does not affect our observations as directly. Further analysis of models similar to ours could clarify when seasonality of infectious diseases could pose a major problem for EWS of disease emergence.

For our model, estimating and removing a periodic trend prior to EWS analysis did not improve prediction uniformly among statistics. This was not entirely surprising because the seasonal signal was not apparent in many time series. Rather, sporadically spaced small outbreaks comprised the dynamics. Therefore, periodic detrending and differencing introduced artificial patterns in the time series. In summary, even in systems where transmission is highly seasonal, and a seasonal trend is apparent in a part of the observation window, seasonal detrending is often disadvantageous for forecasting approaches using early warning signals.

In our study, we held constant the period of forcing and other parameters that sometimes exhibit periodicity (e.g., rate of imported infectious individuals, recovery rate, or demographic rates). We also kept the rate of emergence constant among all simulations. A recent study examining the effects that rate of forcing has on the strength of EWS found that trends in EWS were more difficult to assess in systems with faster rates of change [44]. Thus, we would expect that EWS be less reliable for quickly emerging diseases. Here, we focused on periodicity in transmission because it is the driving variable causing emergence and the other parameters (e.g., demographic and recovery rates) are not typically periodic in human populations. Lastly, as is typical in seasonally forced models of infectious diseases [33] and studies of periodicity in natural systems [43], we focused on seasonality using a sine function. However, we showed that our results were largely invariant to different types of seasonality functions (term-time [5], monthly-averaged [28], and square-wave forcing).

Variance and early warning signals based on spectral properties are beginning to emerge as the most reliable indicators of upcoming transitions. In O’Regan and Drake [22], approach to disease endemicity was much more difficult to forecast than elimination but variance was the best predictor in SIR and SIS systems with immigration. In vector-borne disease transmission, elimination was best predicted by variance and coefficient of variation [45]. Additionally, disturbances in food chain dynamics causing trophic cascades (i.e., the release of prey without suppression by predators) were strongly associated with changes in variance and spectral density of population sizes [21]. These experimental and theoretical results are beginning to reveal the most robust, generic indicators across a wide range of ecological and epidemiological systems.

## Conclusions

Forecasting emerging infectious diseases is of tremendous value to public health and society. Advances in surveillance systems and data science have generated the expectation that early warning systems are not only feasible but necessary tools to combat the spread of infectious diseases [46]. Critical slowing down was previously shown to signal disease emergence without seasonal transmission [22]. Here, we showed that critical slowing down also precedes disease emergence with seasonal transmission. Additionally, we found that statistics based on the wavelet spectrum are robust signals of disease emergence. Future research should aim to document critical slowing down in experimental or surveillance data to estimate the potential forecast horizon that early warning signals provide.

## Additional file

Additional file 1 Supplementary analysis showing effects of different forms of seasonal forcing, window size, and window shape on performance of early warning signals. (PDF 151 kb)
