# Supplementary material for: Forecasting infectious disease emergence subject to seasonal forcing
Source: Theor Biol Med Model. 2017 Sep 6;14:17. doi: 10.1186/s12976-017-0063-8 (PMC5586031; doi:10.1186/s12976-017-0063-8)
Supplement: Additional file 1 — Supplementary analysis showing effects of different forms of seasonal forcing, window size, and window shape on performance of early warning signals. (PDF 151 kb) [file 12976_2017_63_MOESM1_ESM.pdf]

Supplement: Forecasting infectious disease emergence in the presence of seasonally forced transmission

Paige Miller[a,b], Eamon O'Dea[a,b], Pejman Rohani[a,b] and John M Drake[a,b]

[a]University of Georgia, Odum School of Ecology, 140 E. Green Street, Athens GA, USA
[b]Center for the Ecology of Infectious Diseases, University of Georgia, Athens GA, USA

June 29, 2017

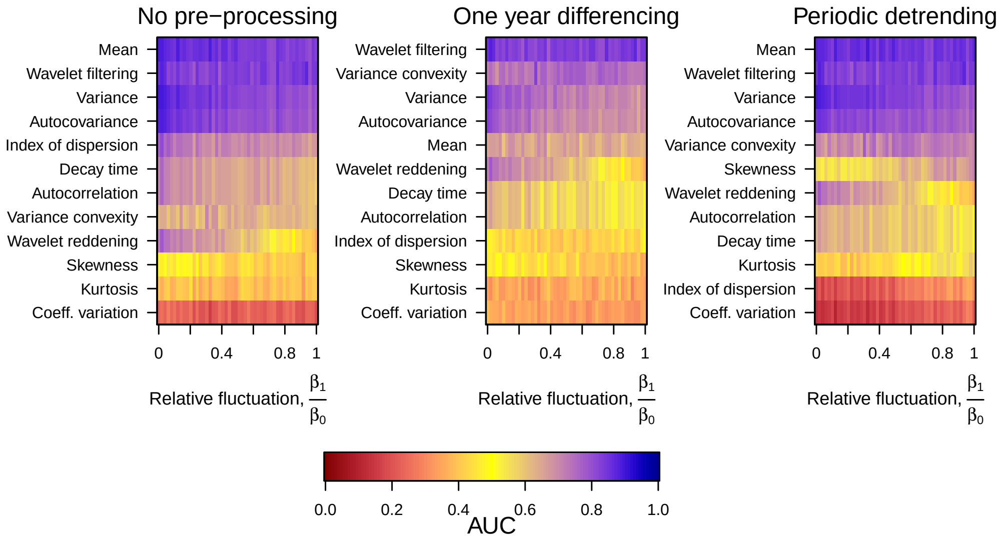

Figure S1: Performance of EWS over a moving window as measured by AUC for SIR systems approaching emergence in the presence of seasonal transmission. The bandwidth for all EWS is equal to 150 weeks. The bandwidth size is not pertinent for wavelet EWS. Data were not pre-processed prior to wavelet-based EWS. All parameters (except for bandwidth) for analyzing EWS are the same as those used in the main text.

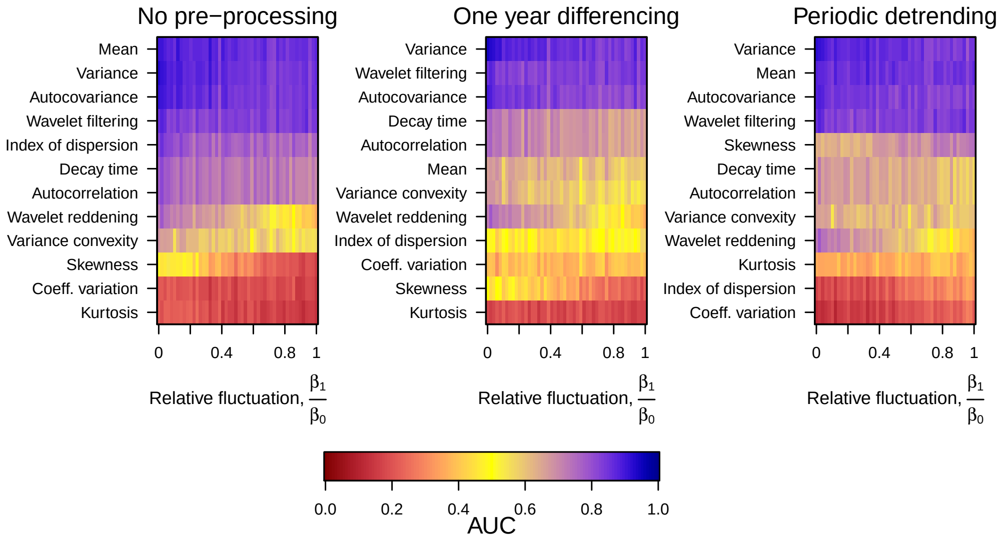

Figure S2: Performance of EWS over a moving window as measured by AUC for SIR systems approaching emergence in the presence of seasonal transmission. Figure shows results using a Gaussian kernel. Data were not pre-processed prior to wavelet-based EWS. All parameters (except for kernel type) for analyzing EWS are the same as those used in the main text.

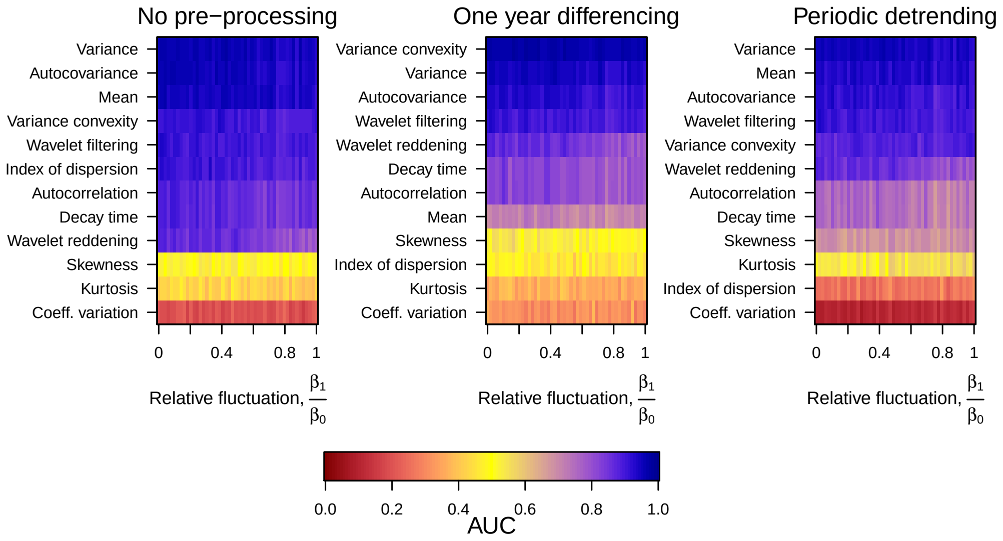

Figure S3: Performance of EWS over a moving window as measured by AUC for SIR systems approaching emergence in the presence of seasonal transmission. Figure shows results with $\beta = 0.02$. Main text shows results for $\beta = 0.04$. Data were not pre-processed prior to wavelet-based EWS. All other parameters for simulating the SIR model are the same as those used in the main text.

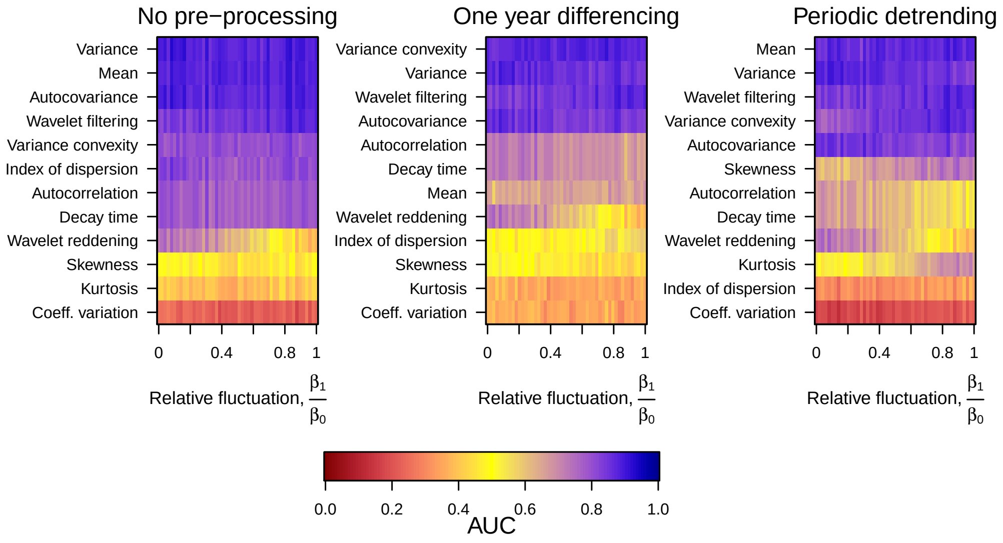

Figure S4: Performance of EWS over a moving window as measured by AUC for SIR systems approaching emergence in the presence of seasonal transmission. Figure shows results when the importation rate is also subject to seasonal (sine) forcing. All other parameters for simulating the SIR model are the same as those used in the main text.

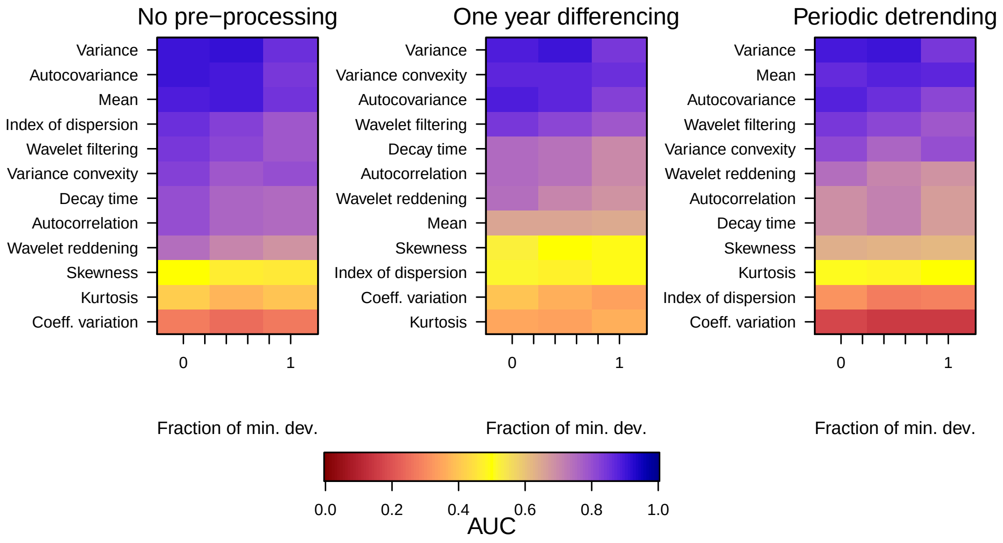

Figure S5: Performance of EWS over a moving window as measured by AUC for SIR systems approaching emergence in the presence of seasonal transmission. Figure shows results when the transmission rate is subject to seasonal (term-time) forcing (Keeling et al., 2001). The average transmission rate remains to be 0.04. See main text for details regarding parame- terization of square wave forcing. All other parameters for simulating the SIR model are the same as those used in the main text.

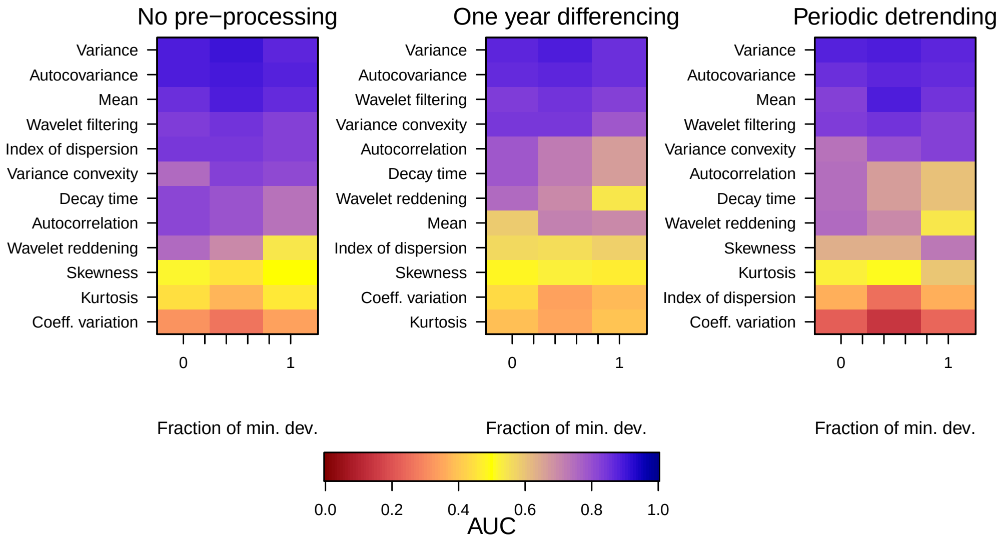

Figure S6: Performance of EWS over a moving window as measured by AUC for SIR systems approaching emergence in the presence of seasonal transmission. Figure shows results when the transmission rate is subject to seasonal (monthly averaged) forcing (Metcalf et al., 2009). The average transmission rate remains to be 0.04. See main text for details regarding parameterization of square wave forcing. All other parameters for simulating the SIR model are the same as those used in the main text.

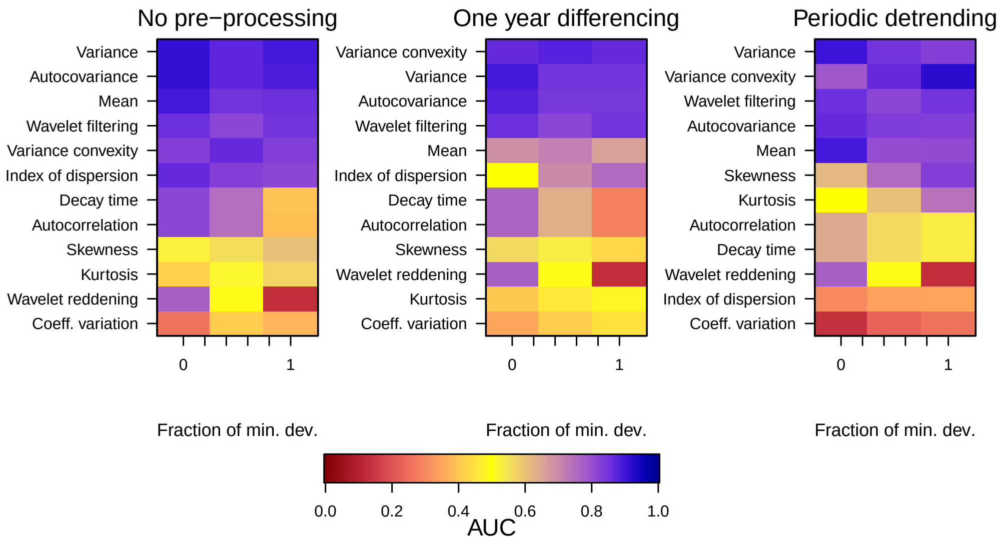

Figure S7: Performance of EWS over a moving window as measured by AUC for SIR systems approaching emergence in the presence of seasonal transmission. Figure shows results when the transmission rate is subject to seasonal square-wave forcing. The average transmission rate remains to be 0.04. See main text for details regarding parameterization of square wave forcing. All other parameters for simulating the SIR model are the same as those used in the main text.
